# Clinicians’ post-operative feeding decisions for infants with congenital duodenal obstruction and trans-anastomotic tube usage: a qualitative interview and focus group study

**DOI:** 10.1186/s12887-026-07310-7

**Published:** 2026-07-14

**Authors:** Alexandra Jager, Joanne Turnbull, Mark John Johnson, Nigel J Hall

**Affiliations:** 1 University of Southampton, University Rd, Southampton , SO17 1BJ UK; 2 School of Health Sciences, University of Southampton, University Rd, Southampton, SO17 1BJ UK; 3 School of Human Development and Health, Faculty of Medicine, University of Southampton, Southampton, UK; 4 Department of Neonatal Medicine, Princess Anne Hospital, Southampton, UK; 5 NIHR Southampton Biomedical Research Centre, University Hospital Southampton NHS Foundation Trust and University of Southampton, Southampton, UK; 6 University Surgery Unit, Faculty of Medicine, University of Southampton, Southampton, UK

**Keywords:** Qualitative, Interviews, Focus Groups, Pediatric

## Abstract

**Background:**

Congenital Duodenal Obstruction (CDO) is a rare birth defect requiring post-surgical nutritional support, using parenteral nutrition (PN), trans-anastomotic tubes (TAT), or both. TATs offer health and cost benefits, but widespread variation in practice exists: most infants receive PN, and few are fed solely via a TAT. This study aimed to understand how and why clinicians make post-operative feeding decisions for infants with CDO focusing on TATs, and to identify requirements for practice change to increase TAT use and reduce PN.

**Methods:**

Clinicians working in paediatrics in the UK’s National Health Service took part in online interviews (*N* = 25) and three subsequent focus groups (*N* = 17 participants). Interviews were coded using thematic analysis to generate themes to understand current feeding practices. Interviewees were sampled purposively to attempt to achieve diversity across workplace location, clinical role, and TAT practice, resulting in participants from 13 different centres and a near-equal distribution of TAT usage: 9 who always or often use TATs, 8 who sometimes use them, and 8 who rarely or never do. Focus groups were used to refine and prioritise interventions for increasing TAT usage.

**Results:**

Interview analysis resulted in four interconnected themes, covering confidence and skills, research scepticism, perceived risks, and organisational inertia. Clinicians requested research demonstrating TATs’ safety and effectiveness that largely already exists in the published literature, suggesting the primary barrier to TAT use is a lack of knowledge translation rather than a lack of evidence. Proposed interventions centred on dissemination of existing research and TAT-specific multidisciplinary education and training.

**Conclusions:**

Clinicians’ reluctance to use TATs is multifaceted, rooted in experiential, organisational, and knowledge-based barriers. Increasing TAT usage does not necessarily require new research: dissemination of existing evidence alongside standardised, multidisciplinary education and care pathways should be the immediate priority.

**Supplementary Information:**

The online version contains supplementary material available at 10.1186/s12887-026-07310-7.

## What is already known on this topic?

- Despite evidence on TATs’ nutrition and cost benefits, there is widespread, unexplained variation in UK post-operative feeding strategies, with most infants receiving PN.
- A previous study found conflicting views among UK-based clinicians regarding TAT risks and effectiveness. Surgeons are usually the sole decision-makers.

## What this study adds

- This is the first study to provide in-depth, rich qualitative insights into the multifaceted reasons for the low uptake of TATs.
- Practical interventions were developed in consultation with clinicians to increase TAT usage.

## How this study might affect research, practice, or policy

- Guide the development and implementation of multidisciplinary care bundles for infants with CDO to reduce reliance on PN, resulting in nutritional benefits and cost savings.
- Inform future research on TATs that addresses gaps in existing research and clinicians’ concerns about TATs’ effectiveness and safety.
- Inform future guidelines on TAT feeding by providing evidence on the barriers that should be addressed to increase this.

## Main article

### Introduction

Congenital duodenal obstruction (CDO) is a rare (1 in 8200 live births) condition typically requiring surgical repair several days after birth [1, 2]. This equates to approximately 110 cases per annum in the UK, of which circa three-quarters undergo duodenoduodenostomy, representing around 3 such procedures per centre per year [2]. After surgery, infants require nutritional support whilst normal gastrointestinal function resumes. Several options for this exist including intravenous feeding using parenteral nutrition (PN), enteral feeding via a trans-anastomotic tube (TAT, which allows feeding into the gut beyond the anastomosis), or a combination of both [1, 3]. Compared to no TAT usage, early enteral feeding via a TAT has been shown to confer benefits including reduced time to feed initiation, fewer days of PN, reduced usage of central venous catheters (CVCs) and their accompanying complications, earlier provision of breast milk and cost savings [4–6]. Despite this, exclusive early enteral feeding via a TAT is rarely used and there is widespread UK variation in postoperative feeding strategies unexplained by demographic and clinical factors. In a large epidemiological study of infants born with CDO, 42% of infants received a TAT following CDO repair, and 88% received PN. Where a TAT was used the infant frequently also received a CVC and PN with only 42% of infants receiving early enteral feeds alone, with the remaining 58% unable to benefit from the advantages associated with early feeding via a TAT [2].

We previously completed a national survey of practice in which neonatal surgeons and neonatologists reported conflicting and wide-ranging views on TATs’ risks and effectiveness, notably citing a lack of supporting research and the perceived risks of TATs as barriers to their (increased) usage [7]. Only 36% of surgeons believed a TAT should always be used. Surgeons were usually (88% of cases) the sole decision-makers about TAT usage. That survey was the building block for this subsequent qualitative study, which provides an in-depth understanding of the barriers to increased TAT usage and outlines actionable, clinician-led interventions to effect change.

Our research questions were:

1. How and why clinicians make post-operative feeding decisions for infants with CDO, to understand TAT usage and the barriers to (increasing) TAT usage (RQ1);
2. What would be required to influence practice change in favour of increased TAT use and less reliance on PN (RQ2).

## Methods

### PPI (Patient and Public Involvement)

Prior to the study commencement, we conducted two online group discussions with parents of infants born with CDO. At the initial meeting existing research evidence was presented, and parents expressed they believed TATs had clear benefits over feeding with PN. They therefore did not feel that a further clinical study, e.g. a randomised controlled trial (RCT), would be acceptable to parents. Instead, they recommended studying barriers to TAT usage using primary data collection to understand better the pathway, including potential barriers, to implementation.

### Study design, setting, and participants

**Fig. 1:**
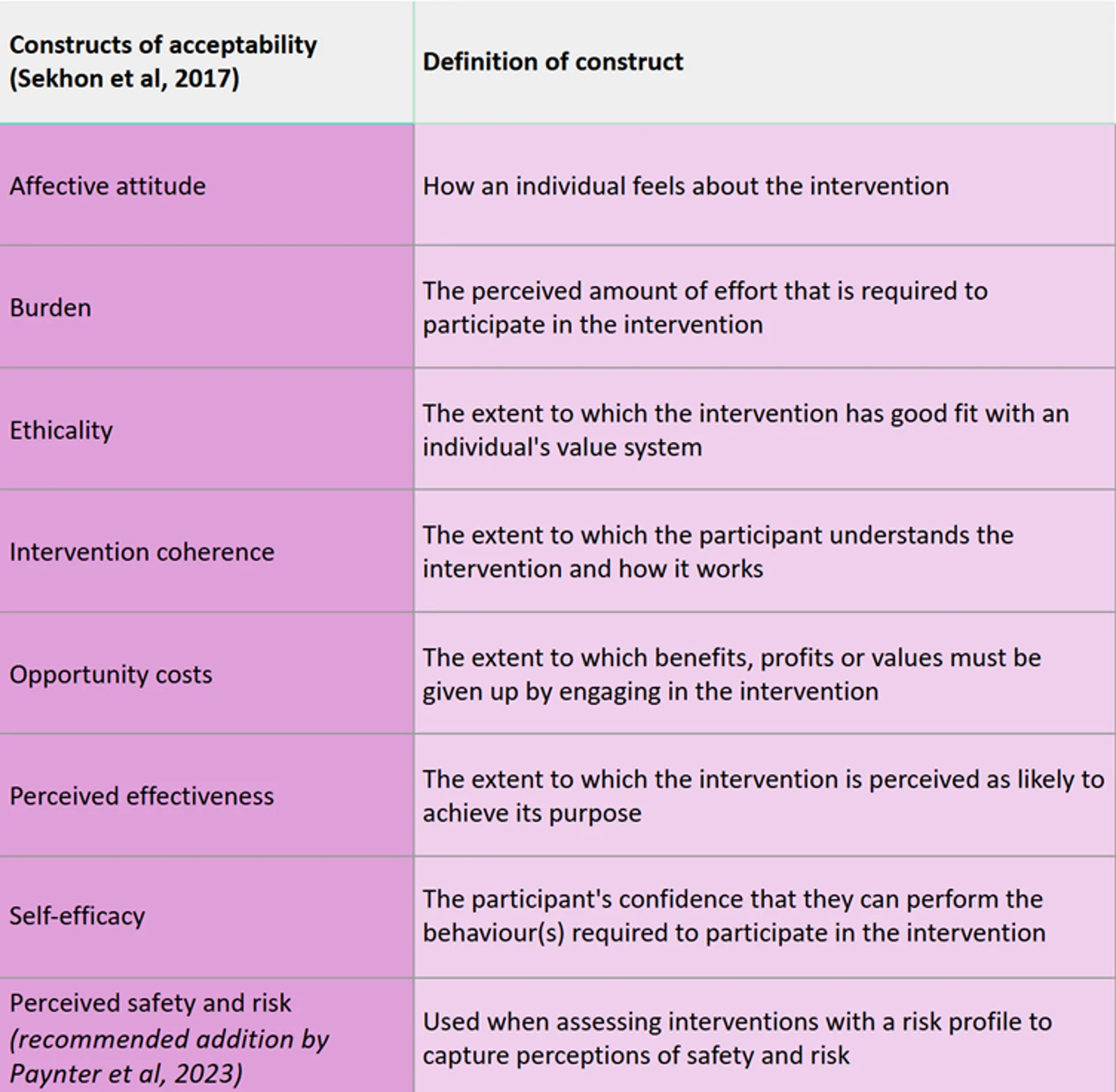
Adapted Theoretical Framework of Acceptability (TFA) domains

We conducted semi-structured interviews and subsequent focus groups with UK-based clinicians experienced in treating infants with CDO. An initial interview guide (supplementary file 1), based on our survey finding [7], was refined iteratively and underpinned by the Theoretical Framework of Acceptability (TFA) domains [8] (including Paynter et al.’s proposed additional construct for surgical settings of ‘safety and risk’ [9]). This ensured comprehensive coverage of intervention acceptability (Fig. 1). We employed a sequential explanatory design, whereby a quantitative survey was followed by qualitative interviews, and later focus groups. The latter qualitative phases (reported in this paper) were used to explain and elaborate on the findings of the first phase (the initial survey also included some open-text questions which were analysed descriptively) [10].

Interviews initially focused on RQ1, but interviewees also noted requirements for practice change (i.e. increased TAT usage, RQ2). Synthesising interview and survey data, we developed an initial ‘long list’ of interventions to increase TAT usage for presentation in the focus groups (supplementary file 2). Participants prioritised interventions and discussed how and why they could work (RQ2). The focus groups drew upon co-design principles, a participatory approach which enables interventions to reflect clinicians’ knowledge of local contexts and needs, thereby increasing adherence and uptake [11, 12]. Consistent with our co-design approach, focus group moderators actively contributed to discussions; while they did not share their own views on the subject, they did inject relevant evidence into discussions. Given that two moderators were clinicians with expertise in this area, complete neutrality would have been difficult to achieve. Study participants were UK-based clinicians with experience of caring for infants with duodenal atresia recruited using various channels, including newsletters of relevant clinical and professional organisations, social media, personal contacts of the authors, and prior survey respondents who had opted in to being contacted. Interviewee recruitment was purposeful to achieve diversity in workplace location, clinical roles, current feeding practices (specifically, regular use of a TAT and not), and years of experience. However, there was a high number of surgeons in the first two focus groups, likely because they are the main decision-makers about TATs [7]. Consequently, a third focus group was conducted with neonatologists only to mitigate professional bias; this group also gave neonatologists a space to speak freely and candidly about inter-specialty dynamics and challenges (especially with surgeons). Interviews and focus groups were conducted using Microsoft Teams, audio recorded, and professionally transcribed and anonymised. This study adheres to Consolidated Criteria for Reporting Qualitative Research guidelines [13] (supplementary file 3).

### Data analysis

Interview and focus group transcripts were analysed using Braun and Clarke’s thematic analysis approach [14, 15] to answer RQ1 using NVivo to support data management and coding [16]. Transcripts were initially read by AJ and initial codes were generated. These codes were mainly semantic, i.e. based on the data’s explicit meanings [15]. Code development was deductive (mainly derived from the survey findings and TFA domains). AJ also led the survey analysis, so they did not start with a ‘clean slate’. New codes were also developed inductively. Codes were grouped into themes and subthemes; once overarching themes had been constructed, additional latent codes were developed. These were interpretive codes which aimed to describe underlying assumptions and ideas [15]. We used a team approach to share and interpret data, and build/review emergent themes.

**Fig. 2:**
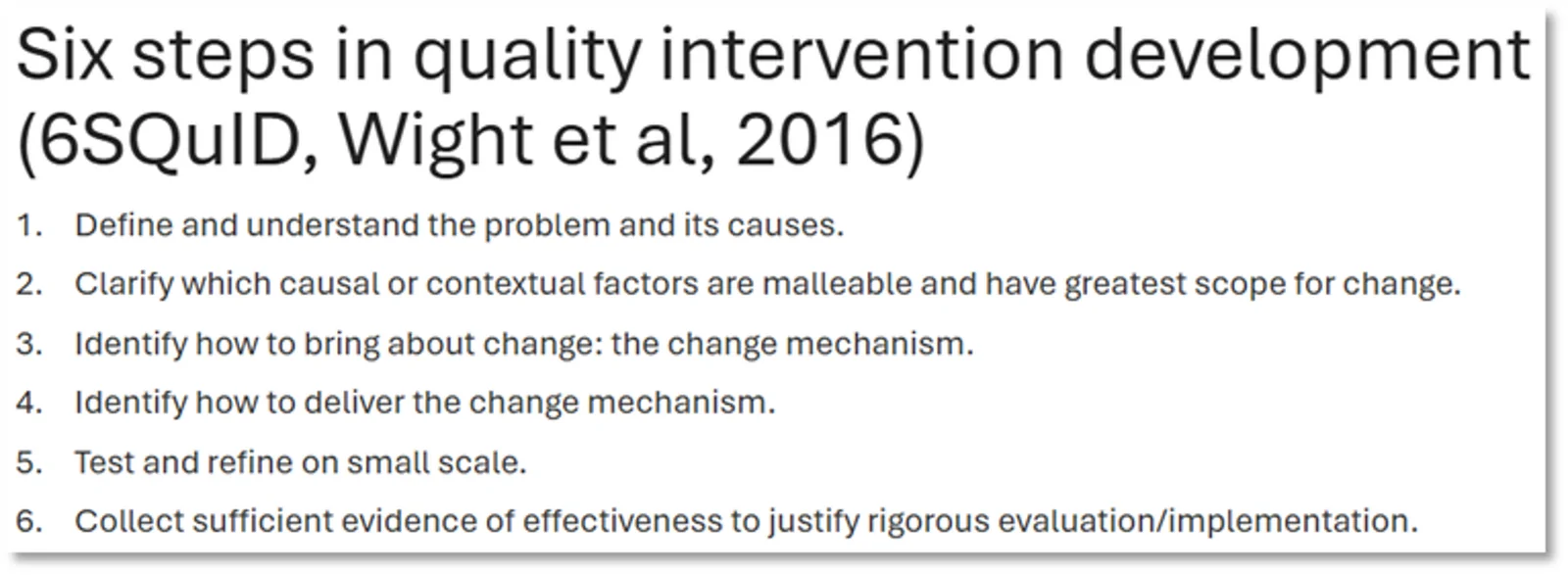
The Six Steps in Quality Intervention Development (6SQuID) framework

Before the focus groups, a draft ‘long list’ (supplementary material 2) of interventions to increase TAT usage was created (RQ2) from interview findings. The interventions were grouped into four themes and presented at the beginning of the focus groups to facilitate intervention development. Participants were asked to choose the most promising interventions from this list or suggest additional ones. In practice, all interventions developed in the focus groups originated from this ‘long list’, i.e. no completely new interventions were proposed. Coding related to intervention development (RQ2) was largely descriptive and summative, undertaken cognisant of steps 2 to 4 of the quality intervention development (6SQuID) framework (Fig. 2), which outlines the essential steps of intervention development in public health [17]. This framework was also used to structure the interventions developed.

## Results

Between July and December 2023 (Table 1), 25 interviews were completed (mean duration: 22 min) with clinicians from 13 different centres (hospitals) (between 1 and 4 clinicians per centre).

**Table 1:** Characteristics of interview participants

	Characteristic	Number
Clinical role	Neonatal / Paediatric Dietitian	1
	Neonatal Surgical Specialist Nurse	3
	Trainee Paediatric Surgeon	3
	Consultant Paediatric Surgeon	14
	Consultant Neonatologist	4
Years of experience in current role	0–10 years	11
	11–20 years	7
	21–30 years	5
	30 + years	2
Current TAT practice	Always or often uses TATs	9
	Sometimes uses TATs	8
	Very rarely or never uses TATs	8

Between January and March 2024 three online focus groups involving 17 clinicians were undertaken, each facilitated by at least two members of the research team (Table 2).

**Table 2:** Focus group participants

	Focus group 1	Focus group 2	Focus group 3	Total number
Duration	80 min	78 min	74 min	
Participants (n):				
Consultant Paediatric Surgeon	4	4	0	**8**
Trainee Paediatric Surgeon	1	0	0	**1**
Neonatal Surgical Specialist Nurse	0	1	0	**1**
Consultant Neonatologist	1	1	5	**7**
Total	**6**	**6**	**5**	**17**

### Post-operative feeding decisions for infants with CDO: themes

#### ‘Can we even use TATs?’ Confidence, skills and resources

Regardless of their current practice, many participants were confident in their ability to use TATs, developed through experience, training, relevant literature, and using feeding tubes to treat similar conditions such as oesophageal atresia. TATs were not considered particularly complex.

A few participants reported lacking skills themselves, whilst others perceived a lack of skills of their colleagues, exacerbated by the rarity of CDO and staff turnover:

*‘Turnover is huge and trying to get them all comfortable is something that probably at ours only happens maybe six times a year. It’s quite difficult.’* (Consultant Paediatric Surgeon 1, Focus Group 1).

Although most participants had access to TATs, a minority did not. Some were unwilling to use the larger and stiffer TATs available to them due to fears of injuring infants, which created difficulty:

*‘The types of the tubes that I have been presented with I think they are a bit more stiff than what I would want to leave as a TAT tube and they are a bit large as well…. [that] would make me really anxious about leading that through the pylorus.’* (Consultant Paediatric Surgeon 22, interview).

#### Are TATs effective? Contradictions, ambivalence, and scepticism about research evidence

Similar to our previous survey findings [7], participants had wide-ranging and contradictory views on the effectiveness and benefits of TATs and PNs. Reported benefits of TATs included quicker time to enteral feeding, including of breast milk, and the avoidance of PN use. Supporters of PN doubted the effectiveness of TATs, believing that they did not lead to earlier feeding or discharge. Some felt that PN use was unavoidable, such that even if a TAT was used it would be inevitable that PN would also be needed.

A few participants viewed the current research on TATs as comprehensive, but many were critical of it and reported a limited influence on their clinical practice, preferring instead to rely on their experience or guidance from colleagues. Some felt current research evidence did not clearly demonstrate TATs’ superiority, suggesting that benefits were statistically but not clinically significant. Others had equipoise about TATs. Some cited limitations of existing research, often completed in single centres, based on too small sample sizes, or had too large confidence intervals. A few highlighted that lack of an RCT meant there was not clear evidence to support TATs. Some believed research could be ‘cherry picked’ to support using or not using TATs. The perceived lack of clear evidence meant that some struggled to translate the benefits reported in research into their own clinical practice.

*‘I think there is some evidence that they can. But I don’t necessarily think that the benefits that are suggested in some of the papers are universally achieved … there are other studies that have found them not to be useful and in some circumstances … actually found them to cause problems.* (Consultant Paediatric Surgeon, interview 4)

*‘There is research out there … but you’ll end up your personal preference. You’ll find the piece of research that supports or you feel comfortable and right with and you’ll use that in your practice.’* (Neonatal Surgical Specialist Nurse, interview 16).

Some participants had limited knowledge of research on TATs, which was felt to be either out-of-date or incomplete:

*‘Off the top of my head when I was preparing for the exams years ago I think it was a meta-analysis that I read somewhere … It must be on the other computer because I’ve got about a thousand papers there but I haven’t actually revised it any time recently.’* (Consultant Paediatric Surgeon, interview 22).

Several participants described conducting their own research on TATs (e.g. reviewing clinical data from their hospital), which influenced their practice more strongly than published research.

#### Are TATs risky? ‘Cortical scars’ and conflicting views

Participants’ views on the risks and of TATs and PN were diverse and broadly in line with the survey findings. TATs’ proponents felt TATs were safe and unlikely to cause complications such as perforations, and were keen to avoid complications associated with CVCs and PN. Conversely, proponents of PN highlighted TAT-specific risks including potential dislodgement, and felt that PN risks were acceptable and manageable.

Several participants described ‘cortical scars’ (i.e. deep-seated emotional trauma or distress that changes a person’s behaviour) due to TAT-related complications during operations they performed, e.g. perforations or having to remove a TAT, which often necessitated further surgery. These events were perceived as catastrophic, upsetting, and traumatic, and often meant the surgeons refused to use TATs again.

*‘People are often influenced by a single event that they experienced…they’ll have one baby who has had a perforation related to a tube. Or perhaps more likely they’ll have heard of a baby somewhere else who once had a perforation related to tube and have decided that’s something they can do about and so therefore never use them… there is a bit of tunnel vision.’* (Consultant Paediatric Surgeon, interview 20).

Some non-surgeons felt that surgeons could shift blame more easily when feeding methods other than TATs were used:

*‘Knowing surgeons*,* they will probably blame it on someone else… So it will be the child got line sepsis because you*,* as a unit*,* didn’t look after that properly. It’s one step removed from their surgical competence.’* (Neonatal / Paediatric Dietitian, interview 1).

Others denied the existence of a ‘blame culture’, but did acknowledge that surgeons were both held and felt more responsible for the failure of TATs than PN.

#### Are TATs worth the effort? Burdens, inertia, and virtuous cycles

In line with participants’ perceptions of TAT vs. PN effectiveness and risk, their views on the burdens of feeding methods varied. Some found TATs easier to use, others preferred PN. ‘Virtuous cycles’ or positive reinforcing feedback loops of TAT and PN usage were described. When a unit adopted one feeding method, clinicians’ confidence and comfort with it grew, good outcomes were achieved, and thus the unit kept using it. The effort required to change a feeding method was often deemed too great, leading to inertia:

*‘…having done it [using TATs] for years and not had a problem why would he go back to sweating over putting in long lines again and giving parenteral nutrition … If it works and once you’ve done it*,* it’s going to be difficult to go back.’* (Consultant Neonatologist, interview 14).

This inertia stemmed also from uncertainty about which feeding method was better. Some participants felt equipoise or ambivalence towards TATs, unsure of the ‘correct’ approach:

*‘I’ve kind of felt*,* given everything in the balance that I haven’t seen an awful lot of point in using them. But I haven’t felt terribly strongly about it.’* (Consultant Paediatric Surgeon, interview 21).

Some felt increasing TAT usage would be difficult due to surgeons’ unwillingness to change:

*‘I think they’re [surgeons] quite strong characters and we’ve got a couple of particularly strong surgeons that their way is the way. Full stop.’* (Neonatal Surgical Specialist Nurse, interview 16).

This perceived ‘strong mindedness’ led to some participants feel it was futile to encourage surgeons to use TATs. However, whilst the decision to use a TAT was usually made by surgeons, sometimes neonatologists decided in isolation to use PN, making TAT usage less likely:

*‘…our neonatal team run lines*,* they make the decisions about lines…I very rarely ask or discuss whether to put a line in*,* it happens by rote as soon as they deem…But most of the time that’s on autopilot because lines are so much a part of their practice. So I can’t guarantee if I put a TAT tube in I wouldn’t come back the next day and find the patient has a line in…’* (Consultant Paediatric Surgeon 6, Focus Group 2).

Conversely, ‘everyone being on board’ with TATs made PN usage less likely, as neonatologists were confident in (sole) enteral feeding.

### Interventions to increase TAT usage

Focus group participants developed two categories of interventions to increase TAT usage (Table 3).

**Table 3:** Proposed interventions to increase TAT usage that were discussed during focus groups

Type of intervention:	How could this intervention be implemented? Requested and suggested features	Requested/discussed in:			Implementation timeframe			Sample quotes
		FG1	FG2	FG3	Immediate	Shorter term (1–3 months)	Longer term (1 year+)	
Creation and dissemination of research	**Methodologies research should employ**:							*‘Well we don’t have TATs at the moment*,* as I say our surgeons are divided. We would need evidence to persuade some of them to do it.’* (Neonatologist, Focus Group 3) *‘People will read an RCT that shows benefit and it if they don’t like to do it they’ll trash the RCT and say it’s inconclusive anyway.’* (Consultant Paediatric Surgeon, Focus Group 1) *‘But I think an RCT would take such a long time…I think you could do an adequate study*,* it might not be perfect*,* but you could do an adequate study in other ways.’* (Consultant Paediatric Surgeon, Focus Group 1) *‘I think the safety data that we would need the most and be most interested in is what it takes to convince our surgeons that it is safe rather than the neonatologists…. So knowing*,* persuading some of our surgeons who are less enthusiastic that there aren’t a preponderance of displaced TATs or anastomotic leaks or perforations from a transanastomotic tube or anything else like that would be the first thing*,* the first piece of data.’* (Neonatologist, Focus Group 3) *‘I think probably RCT would be nice you are going to have to deal with a sceptical probably audience so if you are going to do it you need the evidence to be as robust as it can be’* (Consultant Neonatologist, Focus Group 1) *‘I think probably the key comparator is comparing TAT complications with line complications because if you can prove that TATs have less than lines then you are probably on a winner.’ [Moderator notes that these data already exist.]…Do they. I haven’t looked at any evidence.’* (Consultant Paediatric Surgeon, Focus Group 1) *‘They are basically saying that the effect difference is just probably not worth it and I think no we can’t stand here and I don’t think we can say that it’s going to revolutionise care of babies with duodenal atresia and therefore all of the difficulties with change not even just the ways of changing both people and practice is difficult.’* (Consultant Paediatric Surgeon, Focus Group 2) *‘But it would be good to have*,* I mean neonatal surgery has got so many non-evidenced areas of practice it would be quite nice to be able to have something that was perhaps a little bit more evidence guided.’* (Consultant Neonatologist, Focus Group 1)
	RCT	X	X	X			X (does not currently exist)	
	(Matched) case control study	X					X (does not currently exist)	
	Prospective study	X		X	X (already exist; can be disseminated)			
	(Matched) cohort study	X	X	X	X (retrospective comparative cohort studies already exist; can be disseminated)		X (a matched cohort study does not currently exist)	
	Case series		X				X	
	**Benefits research should demonstrate**:							
	TATs’ safety, including complication rates/adverse events of TATs vs. PN, reduction in sepsis/line infections	X	X	X	X (already exist; can be disseminated)			
	Ability to use TATs in isolation (i.e. without PN)	X	X	X	X (already exist; can be disseminated)			
	TATs’ ability to enable infants to achieve the same or better growth and/or weight gain as PN	X	X	X	X (already exist; can be disseminated)			
	Quicker time to (full) enteral feeding with TATs (vs. PN)	X	X	X	X (already exist; can be disseminated)			
	Quicker time to discharge with TATs (vs. PN)	X	X	X			X (not demonstrated in current research)	
TAT-specific education and training	**All clinicians involved in post-operative feeding need training on their specific TAT duties**	X	X			X		*‘Everybody would have to agree to something and work on it. I think you are absolutely right to start thinking about it as a care bundle and it’s not just about a TAT it’s about how you manage duodenal atresia*,* how you nourish a baby with duodenal atresia post-operatively and get people to work together with a common goal’* (Consultant Paediatric Surgeon, Focus Group 1) *‘A flowchart is much better because of all the different factors that are involved with these types of patients*,* the size*,* whether they’ve got any other comorbidities and things like that. So it just makes it easier and obviously their response to increasing the feeds*,* decreasing the feeds*,* whether you have PN or not have PN alongside TAT tubes and in relation to recycling gastric aspirate at what point.’* (Neonatal Surgical Specialist Nurse, Focus Group 2) *‘Actually the familiarity with TATs not dislodging them*,* managing the gastric aspirates*,* returning them*,* working that out that’s a lot of work and education. We’ve got*,* I can’t remember*,* 50 odd nurses who work on our unit*,* that’s an awful lot of people you need to get that message across to to get the training.’* (Consultant Paediatric Surgeon, Focus Group 2) *‘Even when you move from one*,* not that I’ve done it loads but when you move from one unit to another you tend to do something different to what you did just because that’s the way it is I suppose. So changing that dynamic is really difficult isn’t it and I don’t know what level of evidence you need for that. Clearly you need a good level of evidence but you also need the people who are reading the evidence to believe it and want to change.’* (Consultant Paediatric Surgeon, Focus Group 1)
	**TAT-specific training materials**:							
	Defined care bundles/packages for TATs	X	X			X		
	Guidelines, flowcharts, and pathways for TATs	X	X	X		X		

#### Intervention category 1: creation and dissemination of research

This intervention aims to shift the perceptions of clinicians not yet willing to use TATs. This could be changed via research demonstrating TATs’ safety and effectiveness using methodologies clinicians find credible. Participants defined five safety and nutritional benefits alongside five credible research methodologies (see Table 3). Research had to demonstrate that TATs were at least equal (or superior) to PN. It was noted that existing research already addresses some participant requests, suggesting knowledge gaps and a failure of knowledge translation of existing research.

The necessity for an RCT was contested: some clinicians said they would not change their practice without one. Others deemed an RCT infeasible (due to e.g. required sample sizes) or unnecessary, since demonstrating safety and benefits using other methodologies was possible. However, research is not a panacea; participants noted that some clinicians would not change practice based on any research, even an RCT. Some noted the difference between clinical and statistical significance; demonstration of the latter will not automatically effect change.

#### Intervention category 2: TAT-specific education, training, and promotion

Training must be multidisciplinary (i.e. include surgeons, neonatologists, nurses, etc.) and holistic, focussing on comprehensive nourishment rather than simply telling clinicians how to use TATs. This is essential to increase clinicians’ confidence in their own TAT usage, convince them that their colleagues are competent TAT users, and ensure a unified approach.

This training should reassure neonatologists and surgeons that TATs can be used safely and effectively in isolation (i.e. without PN), as a commonly reported barrier to increased TAT usage was the immediate post-surgical commencement of PN. Requested materials requested included guidelines, flowcharts, and care packages, including guidance on how to treat infants of different weight and gestational age; these materials must be applicable to a multidisciplinary team.

## Discussion

In this qualitative research to explore attitudes related to TAT usage and post-operative feeding practices in infants with congenital duodenal obstruction, we have identified a range of barriers to TAT use and subsequently proposed interventions to overcome these., A key observation is that it appears that research with the potential to effect practice change is either not reaching or is unknown to some clinicians. Clinicians requested research that, for the most part, already exists: four of five requested clinical benefits and two of five requested study methodologies are already documented in the published literature. It appears therefore that the primary barrier to TAT use in CDO may be a lack of knowledge translation, and the immediate priority should be disseminating what is already known.

Other key findings of this study that represent barriers to TAT usage include the concept of ‘cortical scars,’ whereby a single catastrophic experience with a TAT leads to lasting refusal to use them, and the role of organisational inertia in perpetuating PN use. This suggests that increasing TAT usage will require targeted dissemination of existing evidence alongside multidisciplinary education and training to address these experiential and organisational barriers.

### Feasibility and implementation of interventions: next steps

Per the 6SQuID framework, the next step is to pilot and refine interventions on a small scale [17]. Neonatal units are unique, not every one will require the full breadth of all interventions. We suggest developing and trialling an intervention that can be implemented immediately or quickly in several units where TATs are never or seldom used. Each unit should have clinicians, ideally a joint surgical-neonatal dyad, responsible for implementation and monitoring. Some resources (e.g. training materials) could be shared between units. The final step is an evaluation of intervention effectiveness (based primarily on clinical but also cost outcomes) to determine the feasibility of a wider rollout.

#### Intervention category 1: creation and dissemination of research

Participants reported a knowledge gap concerning existing TAT research, suggesting that dissemination of current research findings alone could be sufficient to change practice rather than designing and performing additional research. Such a knowledge translation strategy could be implemented immediately at virtually no cost. A dissemination strategy would require collaboration between surgeons and neonatologists.

There was a lack of awareness of existing peer-reviewed research since study methodologies proposed by participants as being necessary to influence their practice already exist. Four of five requested TAT benefits have already been demonstrated in existing research: safety [2, 6] ability to use TATs in isolation [6, 18], TATs’ ability to enable infants to achieve the same or better growth and/or weight gain as PN [3, 4], and quicker time to enteral feeding vs. PN [1, 19]. The fifth requested benefit, quicker time to discharge with TATs, has not been documented in a published study but is unlikely to be a benefit of TAT use. Two of the five study methodologies requested already exist as well, namely prospective [2, 20] and retrospective comparative cohort studies [4–6, 21] (although no strictly matched cohort studies exist). It is noted that the citations for benefits and methodologies are examples and not exhaustive. However, at the time of writing, no RCT, (matched) case control, case series or matched cohort studies existed.

While some clinicians demanded an RCT to change practice, the rarity of CDO, lack of equipoise, high costs and parental reluctance make design and implementation of an RCT challenging. Furthermore, as discussed in focus groups (Table 1) it is debatable whether even an RCT that demonstrates benefit would have the desired effect on clinical practice. Therefore, initially focusing on low-cost dissemination of existing research is advised.

However, some knowledge gaps remain where additional clinical data could effect change, e.g. a more detailed description of growth outcomes achievable without PN, which neonatologists cited as a specific barrier, achievable via an observational study. Similarly, an objective assessment of the incidence of TAT related complications would potentially overcome the ‘cortical scars’ of some surgeons. Surgeons cited intestinal perforations related to TAT use as a barrier. However, a recent systematic review did not identify a single case of perforation specifically related to TAT use in infants with CDO and found a similar incidence of need for further laparotomy in infants treated both with and without a TAT [1]. Again, this suggests a failure of knowledge translation.

#### Intervention category 2: TAT-specific education, training, and promotion

Developing and disseminating TAT-specific training materials requires an initial audit of NHS resources. Clinicians experienced in TATs could then enhance current materials or create new, more detailed resources using the formats discussed in the focus groups (e.g. flowcharts, etc.) Units experienced in TATs could share resources with those less experienced. These materials should be presented in training with a multidisciplinary team, making the duties of all involved clinicians clear. Promotion of early enteral feeding via TAT and avoidance of PN should be included in any intervention to promote TAT use, alongside reinforcing the importance of effective communication amongst the multidisciplinary team to achieve this. The observation that many feeding decisions are driven by inertia supports the importance of this.

### Comparison to existing literature

This is the first in-depth qualitative study on UK clinicians’ views on feeding practices in infants with CDO. Previous research suggests surgeons’ decision-making as involving distinct mental processes ranging from intuitive and subconscious to analytical and conscious, with decisions often reached by a combination of each [22, 23]. Surgical intuition is developed via practice, observation, and exposure, allowing for pattern recognition and the ability to make decisions without explicit reasoning [23]. This aligns with the diverse information-seeking and decision-making processes employed by study participants. To increase TAT usage, clinicians must have access to research demonstrating safety and effectiveness, but also intuitively feel and have experienced TATs as beneficial and safe.

Whilst we set our initial interview and focus group topics guides within the explanatory model of the Theoretical Framework of Acceptability (TFA) [8], the relevance of the domains varied. Constructs of ‘ethicality’ and ‘opportunity costs’ had limited influence. Considerations related to the domain of ‘perceived effectiveness’ as well as Paynter et al’s recommended additional construct of ‘perceived safety and risk’ [9] had the greatest influence, suggesting a need to further develop a surgery-specific TFA.

### Strengths and limitations

We used complementary qualitative methodologies to explore in depth clinicians’ reasons for TAT usage and non-usage, building on our previous survey [7] and allowing for deeper insights into this topic not possible due to the survey’s nature. Drawing on the survey, we were able to target interview and focus group questions (i.e. avoid spending too much time of topics the survey had revealed as less pertinent), and create pragmatic and feasible interventions to effect change.

Although we attempted to recruit diverse types of clinicians, only one dietitian and no trainee neonatologists were recruited, meaning some perspectives are missing. In addition, only three neonatal surgical specialist nurses took part, and no bedside NICU nurses, who bear the operational burden of managing TATs and whose buy-in will be critical for care bundles, took part. Self-selection bias means that clinicians with strong views on TATs may have been more likely to volunteer for focus groups, yet it appears our sampling strategy was effective at achieving participants from a range of institutions and with a split of experiences of TAT use. This helps to eliminate any institutional bias that may exist and provides a sample of participants with a complete range of background views. We acknowledge that the range of interventions requested during the focus groups may not be exhaustive, but these were the interventions that arose and were prioritised during discussions.

## Conclusion

Clinicians have diverse and complex views on TATs’ safety and effectiveness based on personal experience, perception of existing research, their unit’s culture and their colleagues’ attitudes. By gaining an in-depth understanding of these views, we are able to propose interventions with clinicians to increase TAT usage. The next step is to implement and evaluate these interventions.

## Supplementary Information

Supplementary Material 1

Supplementary Material 2

Supplementary Material 3

## Data Availability

To protect the anonymity of interviewees and focus group participants and the small number of clinicians eligible to take part in this study, the full transcripts are not publicly available.
