# Supplementary material for: Clinicians’ post-operative feeding decisions for infants with congenital duodenal obstruction and trans-anastomotic tube usage: a qualitative interview and focus group study
Source: BMC Pediatr. 2026 Jul 14;26:807. doi: 10.1186/s12887-026-07310-7 (PMC13520327; doi:10.1186/s12887-026-07310-7)
Supplement: Supplementary file 1 [file 12887_2026_7310_MOESM1_ESM.docx]

**Semi-structured interview guide: sample questions**

| **Starting questions** | - Could you please tell me a bit about your background and your clinical experience with TATs? - What is the current practice of TAT usage where you work?   - Who introduced the practice, what drove the decision?   - How often are TATs used, and in what circumstances? - Are there any written guidelines or protocols in place? What flexibility is there around this, and who has the final say? - If you are not a surgeon, how much influence do you have over TAT usage? Do you think different types of clinicians feel differently about TATs? - (For non-surgeons) How do you think surgeons make decisions around TAT usage – what motivates them? |
| --- | --- |
| **Affective attitude** | - How do you feel about the practice of TATs? Do you like or dislike it? - What common problems do you (and other clinicians) encounter when using TATs? Does this make you use them more or less? - To what extent are the opinions of parents and carers taken into account? - How might experiencing a catastrophic event/complication during TAT usage influence future TAT usage? How, at all, is this linked to blame culture? |
| **Self-efficacy** | - How confident are you in your abilities to use TATs, and are you comfortable using them? - What factors have helped you build confidence/skills around TATs? |
| **Intervention coherence** | - How do you rate your knowledge and understanding of TAT feeding? - Where and how did you obtain/build this knowledge? |
| **Burden** | - What is the effort required to use/initiate TATs for different types of clinicians? - To what extent do you have the resources required to use TATs? - Do cost factors play a role? |
| **Ethicality** | - Do you feel there are any moral or ethical challenges related to using TATs? - Can they be used fairly? |
| **Opportunity costs** | - Do you feel there are any opportunity costs around using TATs? Does their usage ever conflict with other priorities? - Are there any trade-offs to be made when using TATs? |
| **Perceived effectiveness** | - What are your perceptions of the efficacy of TATs compared to the alternatives? - Which metrics or outcomes do you consider important when deciding to use TATs? - What types of research or evidence have you consulted about TATs? What type of research is or would be the most effective? (Potential follow-up: what would happen if no RCT on this topic materialises?) |
| **Safety & risk** | - What do you consider to be the biggest risks associated with TAT usage vs. PN? |
| **Closing questions** | - What do you think your future practice will look like? Do you think TATs will be used more or less often, and why? - What things would persuade you (and others) to adopt the practice / use the practice more? - Is there anything you would have liked to discuss today that we didn’t? |
