# Supplementary material for: Clinicians’ post-operative feeding decisions for infants with congenital duodenal obstruction and trans-anastomotic tube usage: a qualitative interview and focus group study
Source: BMC Pediatr. 2026 Jul 14;26:807. doi: 10.1186/s12887-026-07310-7 (PMC13520327; doi:10.1186/s12887-026-07310-7)
Supplement: Supplementary file 2 [file 12887_2026_7310_MOESM3_ESM.docx]

**Consolidated criteria for reporting qualitative studies (COREQ): 32-item checklist**

Developed from:

Tong A, Sainsbury P, Craig J. Consolidated criteria for reporting qualitative research (COREQ): a 32-item checklist for interviews and focus groups. *International Journal for Quality in Health Care*. 2007. Volume 19, Number 6: pp. 349 – 357

| **No. Item** | **Guide questions/description** | **Reported on Page # / additional information** |
| --- | --- | --- |
| **Domain 1: Research team and reﬂexivity** |  |  |
| *Personal Characteristics* |  |  |
| 1. Inter viewer/facilitator | Which author/s conducted the interview or focus group? | All interviews were conducted by AJ. The focus groups were moderated by AJ, MJ, and NH. |
| 2. Credentials | What were the researcher’s credentials? E.g. PhD, MD | AJ: DPhil, MSc, BA  JT: PhD  MJ: PhD, BM, BSc  NH: PhD |
| 3. Occupation | What was their occupation at the time of the study? | AJ: postdoctoral mixed methods health services researcher  JT: Associate Professor in health services research  MJ: academic neonatologist  NH: academic paediatric surgeon |
| 4. Gender | Was the researcher male or female? | AJ & JT: female  NH & MJ: male |
| 5. Experience and training | What experience or training did the researcher have? | All four researchers have postgraduate degrees and experience of completing qualitative and mixed methods research. AJ & JT are non-clinical health services researchers. MJ is a qualified neonatologist, and NH is a qualified paediatric surgeon. |
| *Relationship with participants* |  |  |
| 6. Relationship established | Was a relationship established prior to study commencement? | Participants were sent an information sheet prior to the interviews and focus groups. AJ introduced herself at the beginning of the interviews, and interviewees were given the option to ask questions prior to commencing. All the focus group participants briefly introduced themselves before the discussion began. Some of the focus group participants already knew NH and MJ due to their clinical practice. |
| 7. Participant knowledge of the interviewer | What did the participants know about the researcher? e.g. personal goals, reasons for doing the research | The information sheets shared before the interviews and focus groups provided a summary of the research project and interviewer’s job title.  Participants were given the option of asking questions or raising concerns before commencing the interviews. |
| 8. Interviewer characteristics | What characteristics were reported about the inter viewer/facilitator? e.g. Bias, assumptions, reasons and interests in the research topic | See answer to question 5 above.  The focus groups were all attended by either NH or MJ, and some of the attendees were their clinical colleagues. All interviewees and focus group participants were introduced to AJ as a non-clinical postdoctoral researcher. |

| **Domain 2: study design** |  |  |
| --- | --- | --- |
| *Theoretical framework* |  |  |
| 9. Methodological orientation and Theory | What methodological orientation was stated to underpin the study? e.g. grounded theory, discourse analysis, ethnography, phenomenology, content analysis | See section ‘Study design, setting, and participants’. |
| *Participant selection* |  |  |
| 10. Sampling | How were participants selected? e.g. purposive, convenience, consecutive, snowball | See section ‘Study design, setting, and participants’. |
| 11. Method of approach | How were participants approached? e.g. face-to-face, telephone, mail, email | See section ‘Study design, setting, and participants’. |
| 12. Sample size | How many participants were in the study? | See ‘Results’. |
| 13. Non-participation | How many people refused to participate or dropped out? Reasons? | N/A – no one chose to drop out. |
| *Setting* |  |  |
| 14. Setting of data collection | Where was the data collected? e.g. home, clinic, workplace | All remote. |
| 15. Presence of non-participants | Was anyone else present besides the participants and researchers? | No. |
| 16. Description of sample | What are the important characteristics of the sample? e.g. demographic data, date | See ‘Results’. |
| *Data collection* |  |  |
| 17. Interview guide | Were questions, prompts, guides provided by the authors? Was it pilot tested? | See supplementary files 1 and 2 and ‘Study design, setting, and participants’. |
| 18. Repeat interviews | Were repeat interviews carried out? If yes, how many? | Every interviewee was interviewed only once. However, there was some overlap between the interviewees and focus group participants. |
| 19. Audio/visual recording | Did the research use audio or visual recording to collect the data? | Interviews were audio recorded. Focus groups were video recorded to facilitate transcription. Only a professional transcriber had access to the recordings. |
| 20. Field notes | Were ﬁeld notes made during and/or after the interview or focus group? | During the interviews, AJ sometimes took notes about potential follow up questions and further probes. These were not included in the formal analysis. |
| 21. Duration | What was the duration of the interviews or focus group? | See ‘Results’. |
| 22. Data saturation | Was data saturation discussed? | See ‘Strengths and limitations’. |
| 23. Transcripts returned | Were transcripts returned to participants for comment and/or correction? | No. |
| **Domain 3: analysis and ﬁndings** |  |  |
| *Data analysis* |  |  |
| 24. Number of data coders | How many data coders coded the data? | See ‘data analysis’. |
| 25. Description of the coding tree | Did authors provide a description of the coding tree? | The four overarching themes are provided in the results section. The entire coding tree in NVivo is available to the research team. |
| 26. Derivation of themes | Were themes identiﬁed in advance or derived from the data? | See ‘data analysis’. |
| 27. Software | What software, if applicable, was used to manage the data? | NVivo, Microsoft Word. |
| 28. Participant checking | Did participants provide feedback on the ﬁndings? | No. |
| *Reporting* |  |  |
| 29. Quotations presented | Were participant quotations presented to illustrate the themes/ﬁndings? Was each quotation identiﬁed? e.g. participant number | See ‘Results’. |
| 30. Data and ﬁndings consistent | Was there consistency between the data presented and the ﬁndings? | Yes |
| 31. Clarity of major themes | Were major themes clearly presented in the ﬁndings? | See ‘Results’. |
| 32. Clarity of minor themes | Is there a description of diverse cases or discussion of minor themes? | See ‘Results’. |
